# First description of the male caste of the Himalayan endemic ant *Lasius alienoflavus* Bingham, 1903 (Hymenoptera: Formicidae), with re-description of the female and queen castes

**DOI:** 10.3897/BDJ.2.e1136

**Published:** 2014-08-22

**Authors:** Himender Bharti, Irfan Gul

**Affiliations:** † Punjabi University, Patiala, India

**Keywords:** Ants, taxonomy, Male caste, Formicidae, Himalaya

## Abstract

The present paper provides a description of the male caste and re-description of the worker and queen castes of the poorly known ant species *Lasius alienoflavus* Bingham, 1903. This species has hitherto been reported only from the Himalayas, and the present data are also based on specimens collected in the north-western part of the mountain range. Likewise other Himalayan ants, this species also shows restricted distribution, which suggests a rather high degree of endemism (45%) of this group in the Himalayas.

## Introduction

The genus *Lasius* Fabricius, 1804 is hithrto known to comprise 111 extant species and 3 subspecies (Bolton 2014). In the Himalayas, 17 species have so far been reported, including 13 species from the Indian Himalaya (Bharti 2008, Bharti and Gul 2013). A revision of *Lasius* was published by Wilson (1955), where the author placed several species, i.e. *Lasius flavus* (Fabricius, 1804), *Lasius nearcticus* Wheeler, 1906 *Lasius alienoflavus* Bingham, 1903, *Lasius talpa* Wilson, 1955 and *Lasius fallax* Wilson, 1955 forming the *flavus*-group in a distinct subgenus *Cautolasius* Wilson, 1955, and provided a key to the Palaearctic species. Seifert (1992) revised the subgenus *Lasius* *sensu stricto* from the Palaearctic, describing 17 new species, and updated Wilson's key to the Palaearctic species. Other significant contributions form the Palaearctic region include: Bingham (1903) – key to Indian species; Menozzi (1939) – key to the species of Himalayas and Tibet; Collingwood (1982) – key to the Himalayan species; Kupyanskaya (1989) and Radchenko (2005) – key to the Lasius (Dendrolasius) species of East Palaearctic.

Bingham (1903) described *Lasius alienoflavus* Bingham, 1903 from the Himalayas (above 8000 feet) with reports of worker and queen castes. Later on purely morphological grounds Wilson (1955) erected a distinct subgenus *Cautolasius* and placed *Lasius alienoflavus* in it. This subgenus comprises a group of species showing a mixture of characters that put them in a position intermediate between *Lasius* *sensu stricto* and *Chthonolasius*. Subsequently, Collingwood (1982) discussed the worker and queen caste of *Lasius alienoflavus* and provided a key to the Himalayan members of the genus. In the discussion part he mistakenly assigned the species to the subgenus *Chthonolasius*. Recently Bharti and Gul (2013) provided a key for the genus *Lasius* from the Himalayas.

Here we re-describe the worker and queen castes of *Lasius alienoflavus* based on fresh material and for the first time provide a description of the male caste.

## Materials and methods

The specimens were collected by handpicking. The morphological analysis was carried on with the aid of a Nikon SMZ 1500 stereo zoom microscope. All digital images were taken with a MP evolution digital camera and subsequently processed with Auto-Montage software (Syncroscopy, Division of Synoptics, Ltd.) and Adobe Photoshop CS5. All measurements were recorded in millimeters using oculometer between 50× and 125× to the nearest 0.001 mm and have been rounded to the nearest 0.01 mm (as the average recording error was approximately 0.005 mm). Specimens have been deposited in PUPAC, Punjabi University Patiala Ant Collection, Patiala.

### Measurement

HL – Length of the head measured in full face view in a straight line from the middle of the anterior clypeal margin to the middle of the occipital margin. The head has to be carefully tilted to the position with the real maximum.

HW – Maximum width of the head in full face view, behind the eyes.

EL – Maximum eye length with eye in full face view.

EW – Diameter of eye measured perpendicularly to transect in EL and across structurally defined ommatidia.

SL – Maximum straight-line length of the scape excluding the basal neck and the condyle.

WL – Weber’s length – the length of mesosoma in profile from the margin of neck shield to the posterior margin of propodeal lobes.

PW – Maximum width of the petiole from above, in dorsal view.

GL – The length of the gaster in lateral view from the anterior most point of first gastral segment to the posterior most point (excluding sting if present).

HS – Head size – the total of head length and head width divided by hundred.

nHS – Number of standing hairs projecting > 0.02 mm from dorsal profile of scape i.e. the number of hairs visible when looking at the small diameter of scape under transmitted-light conditions. The always present hairs on distal apex are not counted and the number refers to one scape.

nHHT – Number of standing hairs projecting > 0.02 mm from extensor profile of one hind tibia. The always present hairs on distal apex are not counted and the number refers to one tibia.

TL – The total outstretched length in profile from anterior clypeal margin to the posterior most point of gaster excluding sting.

### Indices

Cephalic Index – CI = HL/HW × 100

Eye Index – EI = EL/HW × 100

Scape Index – SI_1_ = SL/HL × 100

Scape Index – SI_2_ = SL/HW × 100

## Taxon treatments

### Lasius alienoflavus

Bingham, 1903

Lasius alienoflavus Bingham, 1903 – Bingham 1903: 341 (w.q.) India
Lasius (Cautolasius) alienoflavus – Wilson 1955: 111; Collingwood 1982: 290.

#### Materials

- **Type status:** Other material. **Occurrence:** recordedBy: Irfan Gul; sex: 23 workers; **Location:** country: India; stateProvince: North-West Himalaya; verbatimLocality: Himachal Pradesh: Khajjiar; maximumElevationInMeters: 2100; **Event:** eventDate: 2010-07-01; **Record Level:** collectionCode: Insect (Ants)
- **Type status:** Other material. **Occurrence:** recordedBy: Irfan Gul; sex: 63 workers; **Location:** country: India; stateProvince: North-West Himalaya; verbatimLocality: Himachal Pradesh: Kharapathar; maximumElevationInMeters: 2800; **Event:** eventDate: 2008-08-13; **Record Level:** collectionCode: Insect (Ants)
- **Type status:** Other material. **Occurrence:** recordedBy: Irfan Gul; sex: 20 workers, 2 queens, 1 male; **Location:** country: India; stateProvince: North-West Himalaya; verbatimLocality: Himachal Pradesh: Manali; maximumElevationInMeters: 1800; **Event:** eventDate: 2010-06-17; **Record Level:** collectionCode: Insect (Ants)
- **Type status:** Other material. **Occurrence:** recordedBy: Irfan Gul; sex: 3 workers; **Location:** country: India; stateProvince: North-West Himalaya; verbatimLocality: Himachal Pradesh: Sungri; maximumElevationInMeters: 2600; **Event:** eventDate: 2008-08-14; **Record Level:** collectionCode: Insect (Ants)
- **Type status:** Other material. **Occurrence:** recordedBy: Irfan Gul; sex: 62 worker; **Location:** country: India; stateProvince: North-West Himalaya; verbatimLocality: Jammu & Kashmir: Sarthal; maximumElevationInMeters: 3000; **Event:** eventDate: 2010-06-04; **Record Level:** collectionCode: Insect (Ants)
- **Type status:** Other material. **Occurrence:** recordedBy: Irfan Gul; sex: 105 workers, 3 queens; **Location:** country: India; stateProvince: North-West Himalaya; verbatimLocality: Uttarakhand: Gangotri; maximumElevationInMeters: 3000; **Event:** eventDate: 2010-06-04; **Record Level:** collectionCode: Insect (Ants)

#### Description

##### Worker

Fig. 1

**Measurements:** HL 0.82–0.91; HW 0.75–0.86; EL 0.12–0.15; EW 0.09–0.12; SL 0.70–0.80; WL 0.88–1.04; PW 0.20–0.24; GL 0.61–1.05; HS 0.78–0.88; CI 101–109; EI 15–18; SI_1 _80–88; SI_2_ 85–95; nHS+nHHT < 8; TL 2.5–2.8. n = 32.

**Head:** Head roughly rectangular in full face view (CI = 101–109); posterior margin of head straight; posterolateral corners rounded; a few setae present closer to posterior margin of head but not reaching the hind margin of eyes and less denser than in *Lasius elevatus* Bharti et Gul, 2013; lateral sides of head more-or-less parallel, somewhat narrowing anteriorly; anterior clypeal margin broadly convex, clypeal carina absent; lateral clypeal profile convex; eyes almost round, size larger as compared to other species of the same group (EI = 15–18); mandibles triangular, the masticatory margin with 7 to 8 teeth, rarely 9, including denticles; antennae 12 segmented, scape long, distinctly surpassing the posterior margin of head (SI_1_ = 80–88, SI_2_ = 85–95).

**Mesosoma and petiole:** Mesosoma with weakly convex promesonotal dorsum slightly depressed at the suture; propodeal dome rather hemispheric, as high as mesonotum, posterior slope of propodeum somewhat straight; area between propodeal spiracle and metapleural gland without distinct setae; in frontal view petiole with weakly convex sides and rather straight dorsum, in profile with steep and slightly convex anterior face and straight posterior face; gaster more-or-less ovate.

**Sculpture and pilosity:** Head and mesosoma with shallow micropunctures; in general body smooth and fairly shiny with scattered pilosity; body covered with suberect to erect setae, abundant and longest on gaster; cuticular surface covered with smooth and rather dense pubescence; genae without standing hairs or setae; scape with subdecumbent to decumbent pubescence, a few setae present at the proximal end; hind tibia pubescence smooth, setae are normally present at the proximal end.

**Colour:** The species is light to dark yellow in colour; the masticatory margin dark brownish and the eyes black in colour; pubescence pale-yellow.

##### Queen

Fig. 2

**Measurements:** HL 1.12–1.15; HW 1.32–1.37; EL 0.34–0.36; EW 0.26–0.27; SL 1.0–1.02; WL 2.33–2.45; PW 0.24–0.26; GL 2.30–2.70; HS 1.24–1.26; CI 81–84; EI 25–26; SI_1 _87–89; SI_2_ 73–74; TL 5.7–6.3. n = 2.

Resembles the worker, with modifications expected for caste and the following differences: body massive, hairy; lateral sides of head subparallel, narrowing towards the anterior margin; eyes much larger; mesosoma enlarged, dorsally flat, scutum and scutellum at the same level, propodeal declivity very steep; in profile view petiole compressed, in frontal view dorsum emarginate; gaster long and gibbous; setae scattered all over and short; head, mesosoma and gaster dark brown, legs dark yellow in colour.

##### Male

Fig. 3

**Measurements:** HL 0.6; HW 0.7; EL 0.27; EW 0.18; SL 0.48; WL 1.3; PW 0.25; GL 1.7; HS 0.65; CI 86; EI 38; SI_1 _80; SI_2_ 68; TL 3.6. n = 1.

**Head:** Head roughly squarein full face view (CI = 86); Head broader at the posterior margin, narrowingtowards the anterior margin; posterior margin of head slightly convex; posterolateral corners rounded; Hairsdenser towards the posterior margin of head; lateral sides of head distinctly narrowinganteriorily; eyes very large, bulging beyond head outline in full-frontal view (EI = 38); three prominent ocelli present at the centre; antennae 13 segmented, filiform; scape long, distinctly surpassing the posterior margin of head (SI_1_ = 80; SI_2_ = 68); clypeus smooth, without any carina in the middle; anterior clypeal margin broadly convex; lateral clypeal profile convex; mandiblewith an apical tooth and 3 denticles at the centre of masticatory margin.

**Mesosoma and petiole:** Mesosomaenlarged to accommodate flight muscles; pronotum small; scutum smooth; scutellum somewhat raised; declivity steep; in frontal view petiole dorsallyround; gaster lengthened, tapering towards the apex.

**Sculptureand pilosity:** Ingeneral body smooth with scattered pilosity; body covered with suberect toerect setae, abundant and longest on gaster; cuticular surface covered withsmooth and rather dense pubescence abundant towards the apex of gaster, antennaeand legs; Hairs more dense on the posterior margin of head, sparselydistributed over rest of the head; standinghairs or setae on genae sparse; mandibles with a few setae at the apicalportion; a few setae present on the anterior margin of clypeus; small blunthairs thinly present on the eyes; scape with subdecumbent to decumbentpubescence, a few setae present at the proximal end; hind tibia pubescencesmooth, setae are normally present at the proximal end.

**Genitals:** Paramereselongated, roughly triangular, covered with long setae; cuspi with short peg-like teeth and bent towards digiti; digiti straight and long, about 3 timesas long as cuspi with round dorsum; penis valve projecting.

**Colour:** brown with a blackish tinge; eyes black; legs and wings creamy-yellowish; body smooth and somewhat shiny all over.

#### Distribution

The overall distribution of the species is from the Himalaya but the specimens used for this study have been collected from the North-west range of Himalaya. All the collection areas were forested mountains surrounded by other mountains. The nests were very close to the surface with a depth of 3–5 inches. The soil surrounding the nest was mainly moist and covered by herbs. 23 specimens were collected from Winkler extraction.

#### Taxon discussion

*Lasius alienoflavus* Bingham, 1903 is well marked off from other reports of this genus and is a distinct species. However, it resembles *Lasius flavus* (Fabricius) but can be readily distinguished from it by having the apical segment of the maxillary palp longer than the preapical one (vs. preapical segment of maxillary palp longer or equal to the apical segment in *Lasius flavus*).

## Supplementary Material

XML Treatment for Lasius alienoflavus

## Figures and Tables

**Figure 1a.**
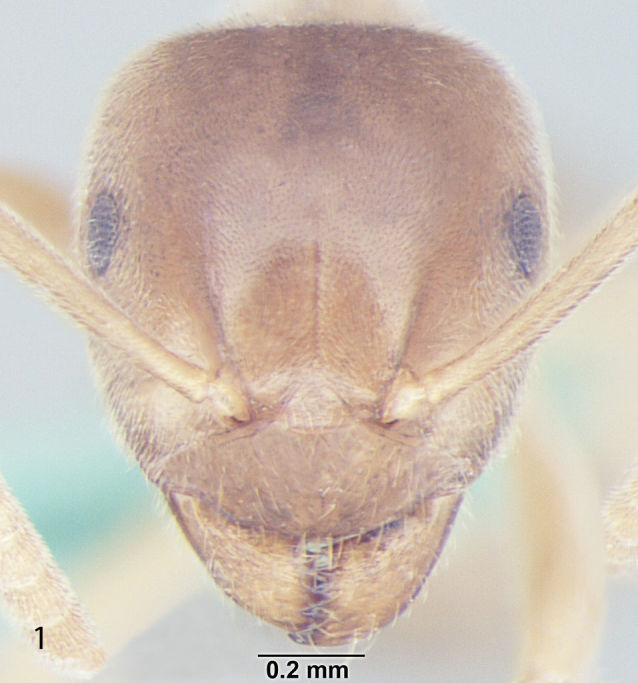
Head, full face view

**Figure 1b.**
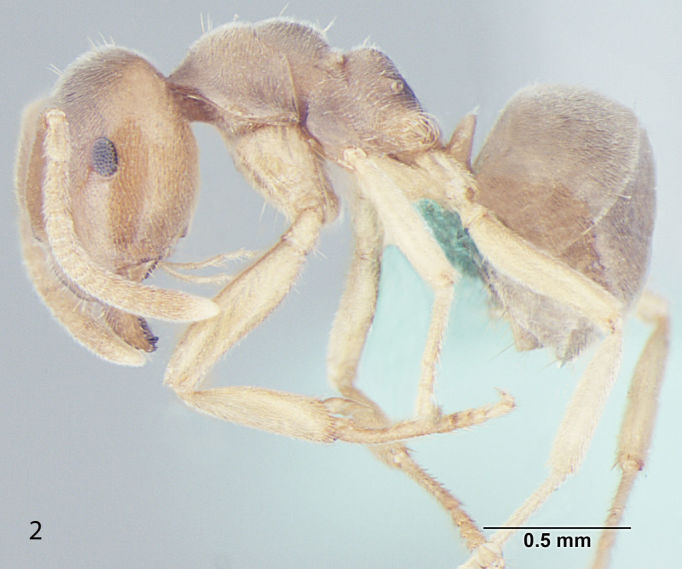
Body, lateral view

**Figure 1c.**
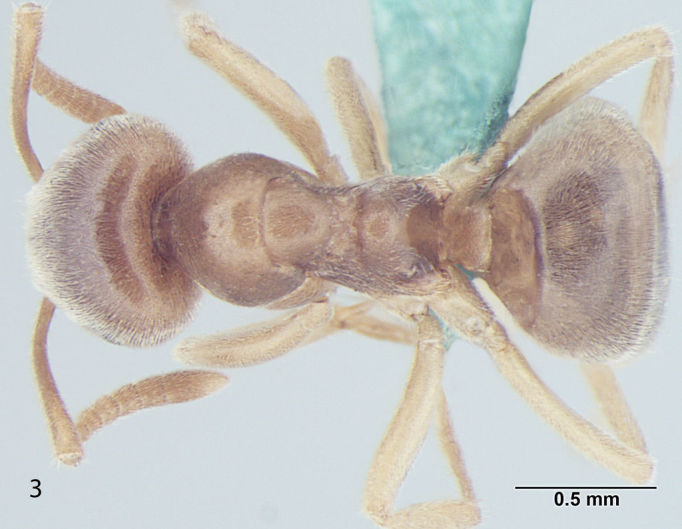
Body, dorsal view

**Figure 2a.**
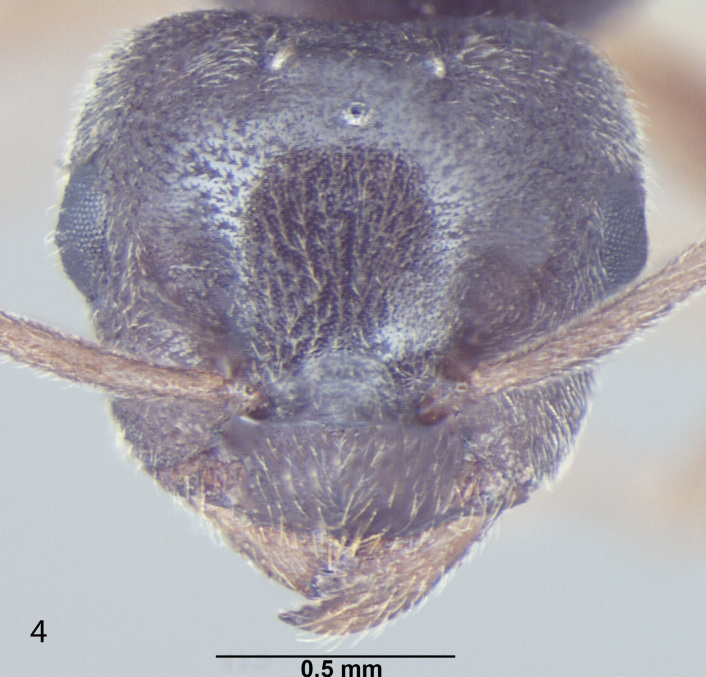
Head, full face view

**Figure 2b.**
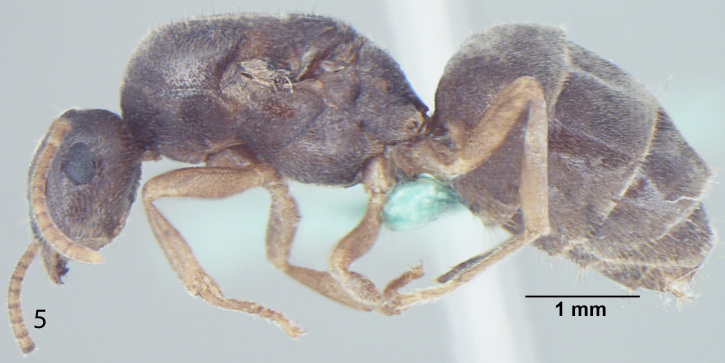
Body, lateral view

**Figure 2c.**
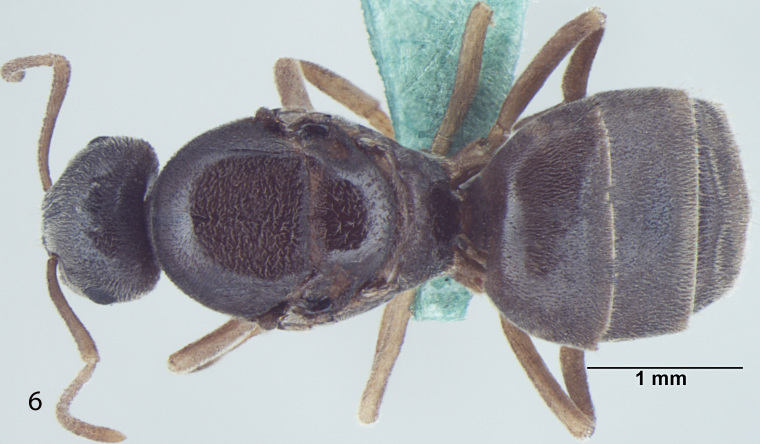
Body, dorsal view

**Figure 3a.**
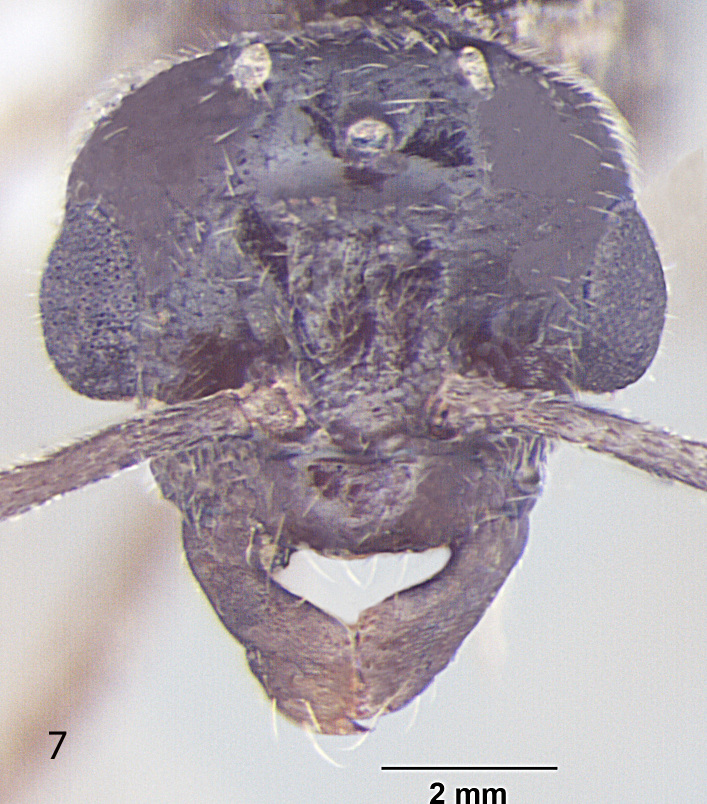
Head, full face view

**Figure 3b.**
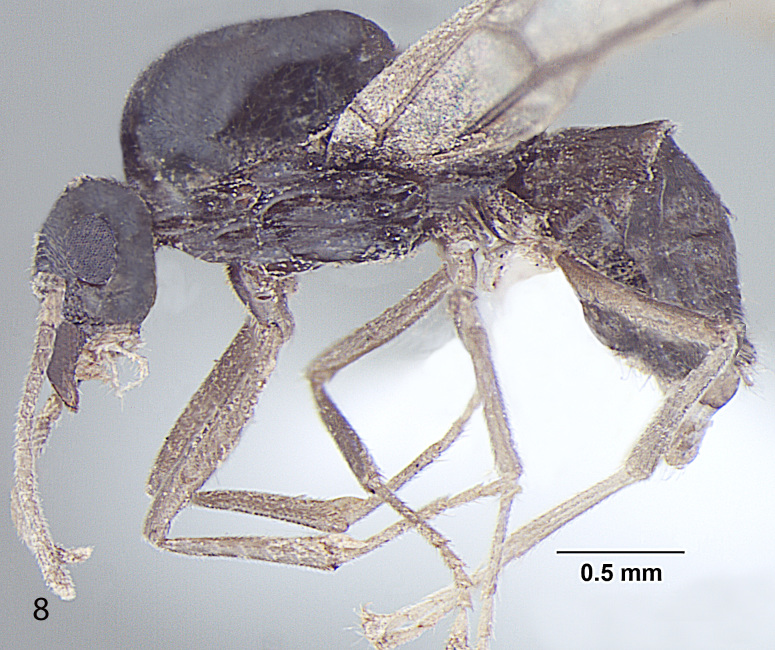
Body, lateral view

**Figure 3c.**
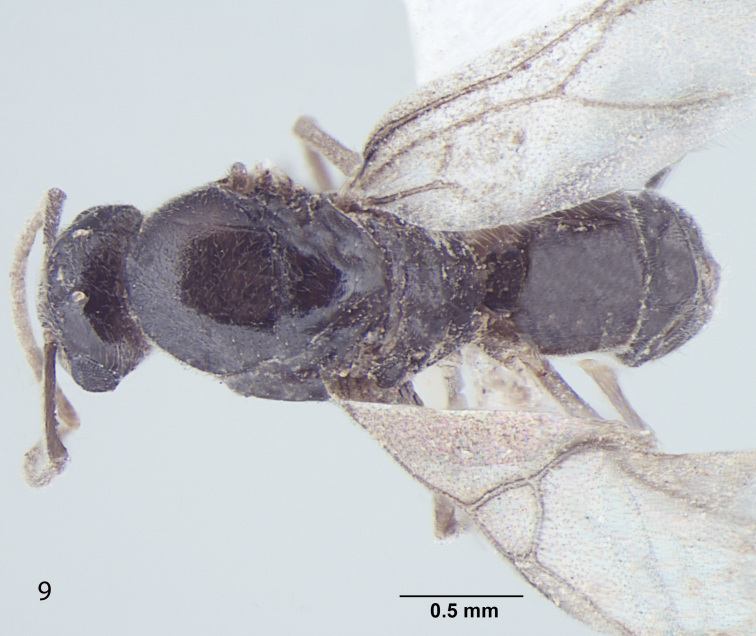
Body, dorsal view
